# Supplementary material for: Outbreak of Western Equine Encephalitis Virus Infection Associated with Neurological Disease in Horses Following a Nearly 40-Year Intermission Period in Argentina
Source: Viruses. 2024 Oct 10;16(10):1594. doi: 10.3390/v16101594 (PMC11512283; doi:10.3390/v16101594)
Supplement: Supplementary file 1 [file viruses-16-01594-s001.zip › viruses-3216794-supplementary.pdf]

Table S1. Primer sequences for conventional PCR and sequencing of nsP4 (derived from Sanchez-Seco et al., 2001 [35]).

Primer name	Sequence (5′ – 3′)
1+ (forward)	GAYGCITAYYTIGAYATGGTIGAIGG
1- (reverse)	CKYTCYTCIGTRTGYTTIGTICCIGG
2+ (forward)	GIAAYTGYAAYGTIACICARATG
2- (reverse)	GCRAAIARIGCIGCIGCYTYIGGICC

Table S2. Primer and probe sequences for EEEV/WEEV-specific RT-qPCR (derived from Lambert et al., 2003 [36]).

Primer/probe name	Sequence (5′ – 3′)
EEEV Fwd	ACACCGCACCCTGATTTTACA
EEEV Rev	CTTCCAAGTGACCTGGTCGTC
EEEV probe	FAM-TGCACCCGGACCATCCGACCT-TAMRA
WEEV Fwd	CTGAAAGTCGGCCTGCGTAT
WEEV Rev	CGCCATTGACGAACGTATCC
WEEV probe	FAM- ATACGGCAATACCACCGCGCACC-TAMRA
